# Corneal thickness and endothelial change after use of ocular hypotensive agents

**DOI:** 10.1002/kjm2.12840

**Published:** 2024-05-28

**Authors:** Hung‐Yin Lai, Hung‐Chi Lai, Ming‐Tse Kuo, Yi‐Yu Tsai, Ing‐Chou Lai

**Affiliations:** ^1^ Graduate Institute of Biomedical Sciences, China Medical University Taichung Taiwan; ^2^ Department of Ophthalmology China Medical University Hospital, China Medical University Taichung Taiwan; ^3^ Department of Ophthalmology Kaohsiung Medical University Hospital Kaohsiung Taiwan; ^4^ Department of Ophthalmology Kaohsiung Chang Gung Memorial Hospital and Chang Gung University College of Medicine Kaohsiung Taiwan; ^5^ Department of Ophthalmology Chiayi Chang Gung Memorial Hospital Chiayi Taiwan

**Keywords:** cornea, glaucoma, ocular hypotensive agents

## Abstract

Corneal transplantation can restore visual function when visual impairment is caused by a corneal disease. However, this treatment is associated with the scarcity of cornea donors. The suitability of corneal donation from patients with glaucoma using ocular hypotensive agents (OHAs) is controversial. This study aimed to elucidate changes in corneal thickness, corneal endothelial cell density, and corneal endothelial cell hexagonality after OHA use in patients with primary open‐angle glaucoma. We retrospectively reviewed the data of 53 glaucoma suspect eyes without OHA use and 106 primary open‐angle glaucoma eyes under OHA use. All participants underwent corneal parameter assessment using SP‐3000P (Topcon Corp., Tokyo, Japan) at the time of diagnosis and the final visit. The OHA dose and timing of use were recorded. The ocular hypotensive agents score (OHAS) was determined based on the number, formula, frequency, and duration of OHA use. Baseline data showed no significant differences between the two groups with and without OHA use. At the final visit, the OHA‐treated group showed significantly lower corneal thickness and corneal endothelial cell density than those of the control group. A weak positive correlation between the OHAS and changes in corneal endothelial cell hexagonality was noted. However, no correlation was observed between the OHAS and changes in corneal thickness or endothelial cell density. In conclusion, patients with glaucoma and using OHAs should undergo the corneal structural properties examinations before donation to ensure the quality of donor cornea.

**Abbreviations**
CAI: carbonic anhydrase inhibitor
IOP: intraocular pressure
OHA: ocular hypotensive agent
OHAS: ocular hypotensive agents score
PGAs: prostaglandin analogues

## 1 INTRODUCTION

Glaucoma is a progressive optic neuropathy characterized by typical structural changes in the optic nerve head and retinal nerve fiber layer, as well as visual function deterioration.^1^ Lowering the intraocular pressure (IOP) with ocular hypotensive agents (OHAs) can suppress glaucoma progression, prevent optic nerve damage, and preserve vision.^2^, ^3^ Most glaucoma cases are controlled by OHAs. However, topical medications for lowering IOP in glaucoma can be toxic to the ocular surface, causing superficial punctate keratitis, corneal erosion, and conjunctival injection.^4^, ^5^ Besides ocular surface damage, previous studies have revealed that patients with glaucoma have thinner corneas than those of individuals without glaucoma.^6^, ^7^ On the other hand, in an eye bank database study, corneal donors with topical glaucoma medication did not show a significant difference in corneal endothelial cell density compared with that of those without topical glaucoma medication.^8^ Whether OHAs influence the structural properties of the cornea remains controversial.

Corneal blindness is a leading cause of blindness worldwide.^9^ Corneal transplantation can restore visual function when visual impairment is caused by a corneal disease.^10^, ^11^ However, cornea donors are limited. With an estimated 12.7 million people waiting for corneal transplantation, only one in 70 of these needs is met worldwide.^12^ Although there have been advancements in artificial corneas in recent years, they have been unable to substitute for donated corneas fully. The demand for donated corneas is significant and indispensable. Patients with glaucoma who face visual disruptions can understand and sympathize with individuals affected by corneal blindness. However, the suitability of corneas for donation remains controversial among patients who use OHAs. While OHA use does not serve as a contraindication for corneal donation, individuals who use OHAs may hesitate to become donors due to potential concerns such as decreased corneal thickness and corneal endothelial cell density.^6^, ^7^, ^8^

Thus, we aimed to evaluate alterations in corneal structural properties in patients with open‐angle glaucoma who used OHAs and to discover the potential correlations between these properties and OHA use.

## 2 MATERIALS AND METHODS

### 2.1 Participants

This study was approved by the Institutional Review Board of the Chang Gung Memorial Hospital, Kaohsiung, Taiwan (Registration Number: 202001462B0), and it adhered to the tenets of the Declaration of Helsinki. This was a retrospective, single‐center study of open‐angle glaucoma and glaucoma suspect. Open‐angle glaucoma was defined by the following criteria: (1) progressive optic neuropathy with characteristic patterns of optic nerve damage and visual field loss, (2) an open, normal‐appearing anterior chamber angle, and (3) elevated IOP.^13^ Patients with glaucoma suspect were identified as those with an open anterior chamber angle exhibiting any of the following characteristics: (1) elevated IOP associated with the normal appearance of the optic disc, retinal nerve fiber layer, and visual field, (2) an optic nerve head or retinal nerve fiber layer showing signs of glaucomatous damage, and (3) a visual field suspicious of glaucomatous damage in the absence of clinical signs of another optic neuropathy or retinopathy.^14^ The clinical records of 159 patients with open‐angle glaucoma or glaucoma suspect diagnosed at the glaucoma clinic of Chang Gung Memorial Hospital were carefully reviewed. None of the patients had corneal anomalies or underwent laser treatment or surgical intervention before or during the follow‐up period. A total of 53 eyes of 53 patients with glaucoma suspect without OHA (controls) and 106 eyes of 106 patients with open‐angle glaucoma treated with OHAs were analyzed. All patients underwent at least two assessments of the corneal structural properties, one at the time of diagnosis and the other at the final visit. The dose and duration of OHA use were also recorded. Annual percentage changes were calculated by dividing the difference in corneal properties (corneal thickness, corneal endothelial cell density, and corneal endothelial hexagonality) by the absolute value of the original values and time in years, scaled by 100.

### 2.2 Parameters of cornea structural properties

Corneal parameters, including corneal thickness, corneal endothelial cell density, and corneal endothelial cell hexagonality, were determined using SP‐3000P (Topcon Corp., Tokyo, Japan). Technicians explained the examination procedure to the participants beforehand, emphasizing the importance of maintaining normal blinking throughout the test. When the center anterior eye segment is aligned to the center of the screen, the auto‐tracking system takes control, focusing precisely on an 8 mm × 8 mm area and automatically centering it. The captured image was transferred to a computer, where the Cell Count Software was used to analyze the data. Corneal thickness, corneal endothelial cell density, and corneal endothelial hexagonality were determined.

### 2.3 Ocular hypotensive agents score (OHAS) determination

The ocular hypotensive agents score (OHAS) was determined for each participant based on the number, formula, frequency, and duration of the OHA used. The OHAS was calculated as follows:

$$\text{OHAS}=\sum_{i=1}^{N}\mathrm{Bi}\times \mathrm{Fi}\times \mathrm{Ti};$$

in which *i* denotes the bottle of the OHA used; N corresponds to 1, 2, or 3 and represents one, two, or three bottles of the OHA used, respectively; *B* corresponds to whether the specified bottle is a single or a compound formula; *F* corresponds to the instillation times per day for this specified bottle; and *T* is the total instillation months for this specified bottle. In addition, participants who had been using medication for less than 6 months were excluded.

### 2.4 Statistical analysis

Only the right eye of each participant was analyzed. All statistical analyses were performed using Microsoft Excel 2016. Using free online statistical software (Social Science Statistics; https://www.socscistatistics.com/), Pearson's correlation coefficient was used to analyze the correlation between OHAS and corneal properties in patients using OHAs. Student's *t*‐tests were used to examine the parametric differences between patients with and without OHA use. Paired *t*‐tests were used to analyze changes in corneal thickness, endothelial cell counts, and endothelial cell hexagonality between the baseline and final visit. Statistical significance was set at *p* < 0.05.

## 3 RESULTS

### 3.1 Clinical profile of patients with and without OHA use

The study included 159 eyes from 159 patients, of which 53 were eyes with glaucoma suspect without OHA use (controls) and 106 were eyes with open‐angle glaucoma under OHA use **(**Table 1 **)**. No significant differences in age, sex, or follow‐up duration were observed between the patients with and without OHA use. At baseline, no significant differences in corneal thickness, corneal endothelial cell density, or corneal endothelial cell hexagonality were observed between the normal control and treatment groups (*p* = 0.32, 0.11, and 0.49, respectively). At the final visit, the OHA‐treated group exhibited significantly reduced corneal thickness and corneal endothelial cell density compared to those of the control group (*p* = 0.01 and 0.01, respectively).

**TABLE 1:** Demographic data of study participants.

Characteristics of participants	Control group	Treatment group	*p*‐value
Age (years)	55.9 ± 17.0	59.6 ± 10.9	0.60
Male (%)	47	60.3	0.11
Follow‐up time (months)	53.2 ± 24.5	61.5 ± 33.10	0.11
Baseline			
Corneal thickness (mm)	0.53 ± 0.03	0.53 ± 0.04	0.32
Corneal endothelial cell density (cells/mm^2^)	2634.17 ± 297.58	2558.57 ± 271.14	0.11
Corneal endothelial hexagonality (%)	54.63 ± 12.46	53.35 ± 9.97	0.49
At final visit			
Corneal thickness (mm)	0.53 ± 0.03	0.52 ± 0.04	0.01*
Corneal endothelial cell density (cells/mm^2^)	2644.14 ± 301.07	2521.96 ± 265.99	0.01*
Corneal endothelial hexagonality (%)	56.13 ± 10.91	53.94 ± 9.97	0.21

*Note*: Control group (without OHA use, *n* = 53); treatment group (with OHA use, *n* = 106). All data were in mean ± standard deviation; *p*‐value was estimated by Student's *t*‐tests.

Abbreviation: OHA, ocular hypotensive agent.

* *p* < 0.05 was considered statistically significant.

### 3.2 Changes in corneal thickness, corneal endothelial cell density, and corneal hexagonality between the baseline and final visit

In the control group without OHA use, no significant differences in corneal thickness, corneal endothelial cell density, and corneal endothelial cell hexagonality were observed between the baseline and final visit (follow‐up time: 53.2 ± 24.5 months). In contrast, in the treatment group with OHA use, no significant differences in corneal endothelial cell hexagonality were observed before and after OHA treatment. However, compared to the baseline, the corneal thickness and corneal endothelial cell density had significantly declined after OHA use (follow‐up time: 61.5 ± 33.1 months) (Table 2).

**TABLE 2:** Changes in corneal thickness, corneal cell count, and corneal hexagonality between the baseline and final visit.

	Baseline	At final visit	*p*‐value
Corneal thickness (mm)			
Control group	0.5320 ± 0.0348	0.5325 ± 0.0360	0.72
Treatment group	0.5256 ± 0.0391	0.5170 ± 0.0371	<0.0001*
Corneal endothelial cell density (cells/mm^2^)			
Control group	2634.17 ± 297.56	2644.14 ± 301.07	0.65
Treatment group	2558.57 ± 271.14	2521.96 ± 265.99	0.02*
Corneal endothelial hexagonality (%)			
Control group	54.63 ± 12.46	56.13 ± 10.91	0.34
Treatment group	53.35 ± 9.97	53.94 ± 9.97	0.61

*Note*: All data were in mean ± standard deviation; *p*‐value was estimated by paired *t*‐tests. Control group (without OHA use, *n* = 53); treatment group (with OHA use, *n* = 106).

Abbreviation: OHA, ocular hypotensive agent.

* *p* < 0.05 was considered statistically significant.

### 3.3 Annual percentage changes in corneal thickness, corneal endothelial cell density, and corneal hexagonality in patients with and without OHA use

Compared with the control group, the treatment group had a significantly greater annual percentage decrease in corneal thickness (*p* = 0.001). However, no significant difference in the annual percentage change in corneal endothelial cell density or endothelial hexagonality was observed between patients with and without OHA use (*p* = 0.22 and 0.31, respectively) (Table 3).

**TABLE 3:** Annual percentage changes in corneal thickness, corneal cell count, and corneal hexagonality.

Annual percentage changes	Control group	Treatment group	*p* value
Corneal thickness (%)	−0.02 ± 0.63%	−0.39 ± 0.70%	0.001*
Corneal endothelial cell density (%)	0.12 ± 1.99%	−0.23 ± 1.55%	0.22
Corneal endothelial hexagonality (%)	1.88 ± 6.08%	0.58 ± 8.04%	0.31

*Note*: Control group (without OHA use, *n* = 53); treatment group (with OHA use, *n* = 106). All data were in mean ± standard deviation; *p* value was estimated by Student's *t*‐tests.

Abbreviation: OHA, ocular hypotensive agent.

* *p* < 0.05 was considered statistically significant.

### 3.4 Corneal property changes with and without carbonic anhydrase inhibitor

**FIGURE 1:**
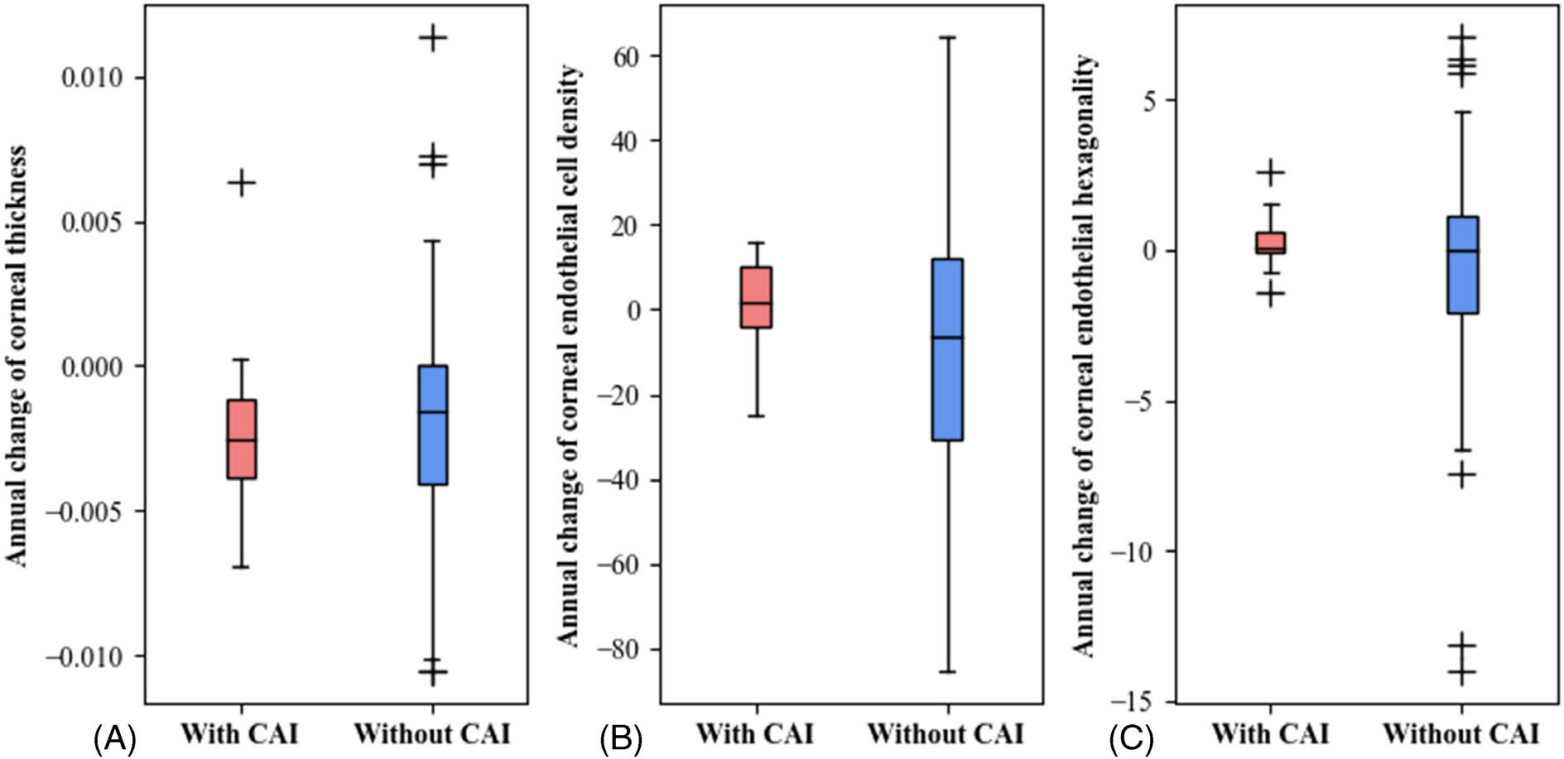
Comparison of annual changes in corneal properties with and without carbonic anhydrase inhibitor use. (A) Annual change of corneal thickness (mm/year). (B) Annual change of corneal endothelial cell density (cells/mm^2^ × year). (C) Annual change of corneal endothelial hexagonality (%/year). Each box was constructed from five parameters, including the median (*Q*2), lower and upper quartiles (*Q*1, *Q*3), and lowest and highest values (*Q*1 − 1.5 × [*Q*3 − *Q*1], *Q*3 + 1.5 × [*Q*3 − *Q*1]). Outliers, outside the lowest and highest values, were marked as +. The *t*‐test was used for statistical analysis. CAI, carbonic anhydrase inhibitor.

Among the 106 eyes with OHA use, 18 patients had topical carbonic anhydrase inhibitor (CAI) formula in their medications, and 88 patients did not. No significant differences were observed in the annual changes in corneal thickness, corneal cell density, or corneal hexagonality between patients with and without topical CAI use (Figure 1).

### 3.5 Corneal property changes with and without prostaglandin use

**FIGURE 2:**
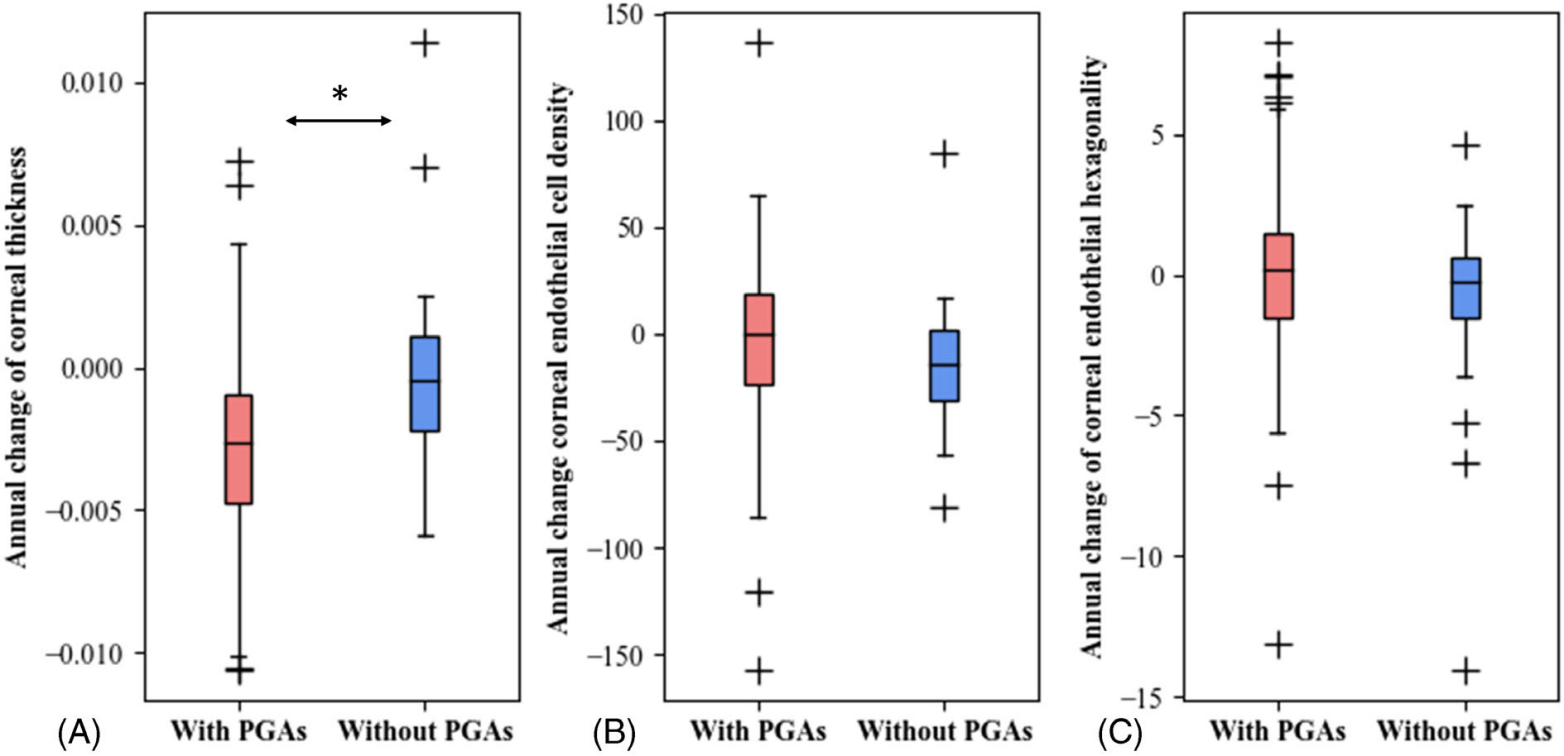
Comparison of annual changes in corneal properties with and without prostaglandin analogues (PGAs) use. (A) Annual change of corneal thickness (mm/year). (B) Annual change of corneal endothelial cell density (cells/mm^2^ × year). (C) Annual change of corneal endothelial hexagonality (%/year). Each box was constructed from five parameters, including the median (*Q*2), lower and upper quartiles (*Q*1, *Q*3), and lowest and highest values (*Q*1 − 1.5 × [*Q*3 − *Q*1], *Q*3 + 1.5 × [*Q*3 − *Q*1]). Outliers, outside the lowest and highest values, were marked as +. The *t*‐test was used for statistical analysis. *p < 0.05 was considered statistically significant. PGAs, prostaglandin analogues.

Among the 106 eyes receiving OHA treatment, 76 patients were prescribed topical prostaglandin analogues (PGAs) in their medication regimen, while 30 patients were not. Patients using topical PGAs demonstrated a significant annual decrease in corneal thickness compared to that in those without PGAs. However, no significant differences in the annual changes in corneal endothelial cell density and corneal endothelial cell hexagonality were observed between patients with and without topical PGAs use (Figure 2).

### 3.6 Correlation between the OHAS and corneal property changes

**FIGURE 3:**
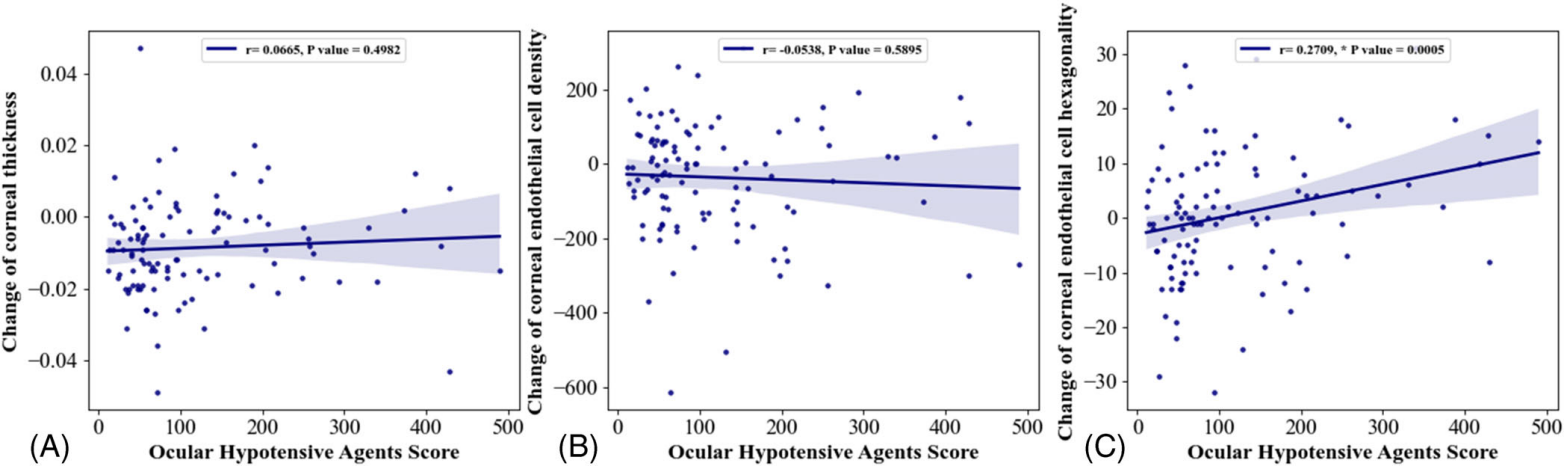
Correlation between the ocular hypotensive agents score (OHAS) and changes in corneal properties. (A) Changes in corneal thickness (mm). (B) Changes in corneal endothelial cell density (cells/mm^2^). (C) Changes in corneal endothelial hexagonality (%). Pearson's correlation coefficient was determined for statistical analysis. OHAS, ocular hypotensive agents score.

Among the patients using OHAs (*n* = 106), no correlation was observed between the OHAS and changes in corneal thickness or corneal endothelial cell density. However, a weak positive correlation was observed between the OHAS and changes in corneal endothelial cell hexagonality (*r* = 0.27, *p* = 0.005) (Figure 3).

## 4 DISCUSSION

Glaucoma is the leading cause of irreversible blindness worldwide.^15^ Topical medications, such as OHAs, are commonly used to reduce the IOP and control glaucoma. However, OHAs may be toxic to the ocular surface and influence the structural properties of the cornea.^4^, ^6^, ^7^ This study demonstrated that OHA use led to a decrease in corneal thickness, and topical PGAs have a significant impact on this reduction. However, the annual percentage change in the corneal thickness was restricted and fell within the range of typical age‐related changes.

In this study, at the final visit, we found a significant decrease in corneal thickness and endothelial cell density in patients treated with OHA. However, when time was considered, only the annual percentage change in corneal thickness significantly decreased with OHA use. This finding is consistent with that of a previous study that demonstrated a statistically significant reduction in central corneal thickness among patients with primary open‐angle glaucoma compared with that in glaucoma suspects.^16^ In addition, a meta‐analysis conducted by Gaspar et al. revealed a significantly lower central corneal thickness in the glaucoma group, in which the decrease in corneal thickness was possibly related to the decrease in corneal stromal thickness.^6^ Furthermore, in a single‐center case–control study, individuals with glaucoma had significantly lower central stromal thickness and central corneal thickness than those of individuals without glaucoma.^7^ Consequently, using an OHA leads to a reduction in corneal thickness, particularly in the stromal components.

However, changes in corneal thickness due to OHA use are limited. Our study showed that the annual percentage change in corneal thickness was approximately −0.39%, which is within the normal range of decrease with aging (−0.1% to −0.7% per year).^16^, ^17^ A previous prospective study reported a reduction of 14 μm in corneal thickness measured with Pentacam® over a 3.6‐year period among healthy individuals (approximately −0.7% reduction per year).^17^ Another longitudinal study revealed the decline in corneal thickness of approximately 5–14 μm across 8.2 years in normal participants (approximately −0.1% to −0.3% reduction per year).^16^ This indicates that patients with glaucoma and OHA use are not a strict contraindication for corneal donation.

We also evaluated the changes in corneal structural properties in patients with and without topical CAI and PGAs use. OHAs, particularly topical CAI and PGAs, alter the structural properties of the cornea. Topical CAI increases corneal thickness, especially in patients with corneal endothelium problems.^18^, ^19^ However, in an 18‐week controlled in vivo experiment, the application of topical dorzolamide did not affect corneal thickness.^20^ Our study yielded comparable findings. We excluded patients with corneal decompensation. We found that topical CAI use did not result in significant differences in the annual changes observed in corneal thickness, corneal cell density, or corneal hexagonality between patients with and without topical CAI use (Figure 1). Thus, the corneal structural properties were not influenced by topical CAI in patients without corneal endothelial problems.

Our study also revealed that patients using topical PGAs had a significant decrease in corneal thickness compared with that of those who did not use topical PGAs. This observation is consistent with the findings of Meda et al., revealing a significant decline in corneal thickness after 6 months of topical PGA use in patients with open‐angle glaucoma.^21^ Additionally, Harasymowycz et al. found that topical PGA treatment significantly reduced the IOP and was associated with central corneal thinning in a 6‐week prospective trial.^22^ Compared to the previous two studies, our study was retrospective, and all patients had used medications for more than 6 months. The short‐term effects may differ from the long‐term effects. Furthermore, meta‐analyses have shown that topical PGAs significantly decrease central corneal thickness.^23^, ^24^ A previous in vitro study showed a decrease in collagen type I levels and corneal thickness in rabbit eyes treated with PGAs and a corresponding decline in corneal thickness in patients treated with PGAs.^25^ In the meta‐analysis, follow‐up duration was not consistent. The different duration made it difficult to determine the age‐related decline in corneal thickness. Topical PGAs can affect corneal thickness, which also changes with age. On considering age, we found a significant difference in annual percentage change with and without PGAs use in corneal thickness (−0.39 ± 0.37% for PGAs use; −0.02% ± 0.34% for non‐PGAs use); both decreases were within the normal range of decrease with aging (−0.1% to −0.7% per year).^16^, ^17^ Therefore, patients using topical PGAs could have experienced decreased corneal thickness to a greater extent than that in those not using PGAs. However, both declines were within the normal range for age‐related changes.

We evaluated the correlation between the OHAS and corneal properties. No correlation was observed between changes in corneal thickness and corneal endothelial cell density. This observation implies that alterations in corneal thickness and endothelial cell density were not associated with the duration or frequency of OHA use. Long‐term use of multiple OHAs in patients with glaucoma is not a strict contraindication for corneal donation.

For corneal donor selection, the eye donor's relevant medical records must be reviewed. Patients with potentially transmissible infectious diseases may be ruled out. In the corneal donor selection, most eye banks accept corneal tissue from donors 2 to 75 years of age, but eye donation at any age is to be encouraged. The minimum acceptable cell count of corneal donation outlined in the eye bank's policy is typically 2000 cells/mm^2^. There are no specific criteria for corneal thickness in the selection of corneal donors, but corneas with increased thickness may be excluded.^26^ Before corneal excision, the cadaveric cornea undergoes inspection. If the cornea exhibits opacity, a central scar, or signs of edematous changes, it would be excluded from corneal donation, as edematous corneas often have increased thickness. In this study, no significant difference in the annual percentage change in corneal endothelial cell density was observed between patients with and without OHA use. Furthermore, all participants had corneal endothelial cell density above 2000 cells/mm^2^ at final visit. This observation implies that patients with glaucoma using OHA is not a strict contraindication for corneal donation.

To ensure the long‐term survival of corneal transplantation, it is advisable to conduct corneal structural properties examinations before donation for patients using OHA. If the donor cornea is transparent and its corneal endothelial cell density is above 2000 cells/mm^2^, meeting the donation criteria, it is suitable for donation. About corneal thickness, there are no specific criteria for corneal thickness in the selection of corneal donors. We found potential pathological changes in corneal thickness associated with the use of OHA. Patients using topical PGAs may experience more reduction in corneal thickness compared to those not using PGAs. However, the changes in corneal thickness fall within the normal range typically associated with aging. Due to limited decline in corneal thickness, it does not affect corneal donation. Thus, patients with open‐angle glaucoma using OHAs, having clear cornea and corneal endothelial cell density above 2000 cells/mm^2^, could donate their cornea.

This study had some limitations. First, this was a retrospective chart review, and the dosage and frequency of medications were based on medical records. However, some patients may have exhibited poor compliance. Second, the corneal structural properties were determined solely based on measurements using SP‐3000P. Previous studies have shown good linear correlations among corneal thickness measurements using different devices.^27^ This suggests that using the same measurement device before and after the follow‐up period is sufficient to assess changes in the corneal structural properties. Third, the participants' data were collected from a single medical center. Further multicenter prospective studies with larger populations are necessary to validate our findings.

In conclusion, the annual percentage changes in corneal thickness was more significant in the OHA‐treated group compared with that in the control group. Nevertheless, this decrease remained limited and fell within the normal range associated with aging. In addition, no significant difference in the annual percentage change in corneal endothelial cell density was observed between patients with and without OHA use. However, a weak positive correlation between the OHAS and changes in corneal endothelial cell hexagonality was noted. Therefore, patients with open‐angle glaucoma using OHAs should undergo the corneal structural properties examinations before donation to ensure the quality of donor cornea.

## CONFLICT OF INTEREST STATEMENT

All authors declare no conflict of interest.
